# Increased mGluR5 in somatostatin-positive interneurons mediates deactivation of the mPFC in a mouse model of neuropathic pain

**DOI:** 10.1038/s12276-025-01435-y

**Published:** 2025-04-10

**Authors:** Mirae Jang, Jaegeon Lee, Seung Ha Kim, Sang Ho Yoon, Myoung-Hwan Kim, Yong-Seok Lee, Sun Kwang Kim, Geehoon Chung, Sang Jeong Kim

**Affiliations:** 1 Department of Physiology, Seoul National University College of Medicine, Seoul, Republic of Korea; 2 Department of Biomedical Sciences, Seoul National University College of Medicine, Seoul, Republic of Korea; 3 Memory Network Medical Research Center, Neuroscience Research Institute, Wide River Institute of Immunology, Seoul National University College of Medicine, Seoul, Republic of Korea; 4 Department of Physiology, College of Korean Medicine, Kyung Hee University, Seoul, Republic of Korea; 5 Department of Science in Korean Medicine, Graduate School, Kyung Hee University, Seoul, Republic of Korea; 6 Department of Physiology, College of medicine, Chungbuk National University, Cheongju, Republic of Korea

**Keywords:** Chronic pain, Neurophysiology

## Abstract

Understanding the neurobiological alterations associated with neuropathic pain is crucial for treatment interventions, but the underlying mechanisms remain unclear. We focused on the medial prefrontal cortex (mPFC), which undergoes various processes of plasticity during the development of neuropathic pain. In particular, in the neuropathic pain state, the pyramidal neuron activity is decreased and metabotropic glutamate receptor 5 (mGluR5) activity is increased in the mPFC. Here we investigated whether mGluR5 inactivation restores neuropathic pain in mice and, if so, how this inactivation affects local circuits in the mPFC. First, we confirmed the analgesic effect of mGluR5 inactivation in the mPFC using a pharmacological approach. Then, via electrophysiological recordings, we showed that the spontaneous inhibitory postsynaptic current (sIPSC) frequency in pyramidal neurons increased during the neuropathic pain state and that this change was attenuated by applying a mGluR5 antagonist. Also, the application of a mGluR5 agonist increased the sIPSC to layer 5 pyramidal neurons in naive mice, consistent with the findings in neuropathic pain conditions. Furthermore, somatostatin (SST)-expressing interneurons in the neuropathic pain group were more depolarized than those in the sham group through mGluR5 activation. Optogenetic inactivation of SST interneurons reversed the increase in sIPSC frequency of pyramidal neurons in the neuropathic pain group. Conversely, mGluR5 overexpression in SST interneurons in the mPFC of naive mice resulted in mechanical allodynia, a representative symptom of neuropathic pain. These results demonstrate that increased mGluR5 activity in SST interneurons contributes to neuropathic pain and that cell-type-specific modulation can provide new avenues for treating neuropathic pain.

## Targeting mGluR5 offers new hope for pain relief

Neuropathic pain is a chronic condition that causes discomfort from normally nonpainful stimuli. Researcher explored how certain brain changes contribute to this pain. They focused on the medial prefrontal cortex, a brain area involved in pain perception. The study examined the role of somatostatin (SST) interneurons, a type of brain cell that inhibits other neurons, in neuropathic pain. Researchers used mice to study how these cells affect pain by manipulating a receptor called mGluR5, which influences neuron activity. They found that activating mGluR5 in SST interneurons increased inhibition of nearby neurons, contributing to pain. Results showed that blocking mGluR5 reduced pain symptoms in mice, suggesting that SST interneurons play a key role in neuropathic pain. The study concludes that targeting mGluR5 in SST interneurons could be a new way to treat neuropathic pain.

This summary was initially drafted using artificial intelligence, then revised and fact-checked by the author.

## Introduction

Neuropathic pain, a type of chronic pain, is often intractable and substantially reduces quality of life. Patients with neuropathic pain experience painful discomfort from stimuli that would not normally cause pain and show increased pain responses^1^. Opioids are currently the most commonly used treatment for pain relief; however, their use is limited by many side effects such as tolerance and addiction^2^. To overcome this neuropathic pain, new treatment addressing the underlying neurobiological factors is required. Previous studies have found associations of neuropathic pain with various supraspinal-level neural alterations, including changes in the brainstem and cortical brain regions^3–5^. Among these, the medial prefrontal cortex (mPFC) is a crucial region for processing pain perception, and various anatomical and functional alterations have been identified in the mPFC of individuals with neuropathic pain^6–9^. In particular, layer 5 pyramidal neurons in the mPFC, which transmit pain-related signals to other pain-related brain regions, exhibit decreased excitability in the neuropathic pain state^8,10–12^. However, it remains unclear which local circuit abnormalities lead to reduced pyramidal activity in neuropathic pain conditions.

Previous studies have reported that the decrease in layer 5 pyramidal neuron output in neuropathic pain state is mediated by increased inhibitory influences^13,14^. During the progression of peripheral nerve injury-induced pain to a chronic state, excitatory inputs to the parvalbumin (PV)-expressing inhibitory interneuron are strengthened, leading to augmented inhibition of pyramidal neuron output^15^. Behavioral experiments have shown that optogenetic activation of PV interneurons lowers the pain threshold^14^. Although most studies have focused on the PV inhibitory neuron, not much is known about the pain-regulating role of somatostatin (SST)-expressing interneurons, another inhibitory cell population known to modulate the activity of pyramidal neurons^16–18^. Recently, it was reported that vasoactive intestinal polypeptide (VIP)-expressing interneurons in the prelimbic cortex show decreased calcium activity in neuropathic pain state and that increasing their activity through chemogenetic modulation alters pyramidal neuron activity and exerts an analgesic effect^12^. Because VIP interneurons are known to predominantly inhibit SST interneurons among other neurons^19,20^, SST interneurons are likely to be disinhibited between VIP and pyramidal neurons modulating pain formation. Also, SST interneurons in the mPFC are known to play a role in working memory by regulating the excitation/inhibition balance, and dysregulation of SST interneurons is associated with neurological disorders such as schizophrenia^16,21^. Based on this understanding, we hypothesized that SST interneurons may also play critical role in the excitation/inhibition imbalance in the mPFC, which is one of the underlying neuroplastic changes associated with neuropathic pain^22,23^. Therefore, we aimed to determine whether the SST interneurons alter the mPFC local circuit in neuropathic pain conditions.

There are reports that the regulation of metabotropic glutamate receptor 5 (mGluR5) on SST interneurons plays an important role in modulating various cognitive behaviors^24,25^. mGluR5 is a Gq-coupled receptor that regulates neuronal excitability and intracellular signal processes^26,27^. In particular, the mGluR5 is expressed on SST interneurons in the mPFC, and the mGluR5 agonist strongly depolarizes SST interneurons^25,28^. Furthermore, previous studies reported that manipulating mGluR5 activity in the brain can regulate neuropathic pain levels^29–31^. In particular, brain imaging data have shown that mGluR5 is upregulated in several brain areas, including the mPFC, in the neuropathic pain state^29^. Therefore, we aimed to confirm whether changes of mGluR5 in the SST interneuron play a role in pain modulation.

The present study investigated how mGluR5 upregulation in the mPFC decreases pyramidal neuron output and modulates neuropathic pain. First, we evaluated the effect of mGluR5 inactivation on neuropathic pain through a pharmacological approach using mGluR5 antagonist 3-((2-methyl-1,3-thiazol-4-yl)ethynyl)pyridine hydrochloride (MTEP). Then, we performed electrophysiological recordings with MTEP or mGluR5 agonist CHPG to determine the effects of mGluR5 modulation on mPFC neurons and to identify the type of interneuron in which mGluR5 activation enhances the inhibition to nearby pyramidal neurons. We also conducted behavioral tests with genetic manipulation to elucidate the effects of modulating mGluR5 on SST interneurons. Our results suggest that SST interneuron-specific mGluR5 activation induces inhibition of pyramidal neurons in the mPFC, which, in turn, is an important mechanism of neuropathic pain.

## Materials and methods

### Animals

All animal experiments were approved by the Institution’s Animal Care and Use Committee of Seoul National University College of Medicine. Four- to five-week-old male wild-type mice (C57BL/6N from Orient Bio) acclimated to the facility for at least 1 week before the experiments. SST-Ai14 and PV-Ai14 mice were generated by crossing either homozygous SST-Cre mice (Sst^tm2.1(cre)Zjh^/J, Jackson Laboratory, #018973) or PV-Cre mice (B6.129P2-Pvalb^tm1(cre)Arbr^/J, Jackson Laboratory, #017320) with homozygous Ai14 Td-Tomato mice (B6;129S6-Gt(ROSA)26Sor^tm14(CAG-td-Tomato)Hze^/J, Jackson Laboratory, #007908). All mice were maintained on a 12-h light/dark cycle with ad libitum access to food and water.

### Behavior test

#### Paw withdrawal thresholds

The paw withdrawal threshold test was used to evaluate mechanical allodynia. It was performed before surgery and more than 7 days after the surgery. The experimenter was blinded to group allocation (neuropathic pain and sham groups). First, during a 5-day habituation period, each mouse was placed under a transparent acryl cage on the grid mesh floor table. Then, on testing days, the mice were acclimated for 30 min before the test began. The right hind paw was exposed to a series of von Frey filaments (2.36, 2.44, 2.83, 3.22, 3.61, 3.84, 4.08 and 4.17) following the up–down method, and the 50% paw withdrawal threshold was determined as previously reported^32^. A withdrawal threshold of 1.4 g was applied as the cutoff. Mice with a sensitive hind paw in the presurgery test were excluded from surgery and subsequent testing, and mice without mechanical hypersensitivity at 7 days after partial sciatic nerve ligation (PSL) surgery were excluded from further experiments.

#### Gait test and ledge test

These tests were applied with a minor adaptation of a previously described method^33^. Mice’s behavioral patterns were evaluated on a scale ranging from 0 to 3, where 3 represents normal behavior and 0 indicates abnormalities. Scoring criteria in the gait test were as follows: 3 for normal movement with full limb support, abdomen off the ground, and even hindlimb participation; 2 for noticeable tremors or limping; 1 for severe tremors, limp, lowered pelvis or misalignment of feet during locomotion (‘duck feet’); and 0 for difficulty moving forward and dragging the abdomen. Subsequently, for the ledge test, mice were placed on the cage’s ledge and observed while walking along it and returning to the cage. Scoring criteria were as follows: 3 for walking along the ledge without losing balance and gracefully lowering into the cage using paws; 2 for occasional loss of footing but overall coordination; 1 for ineffective hind leg usage or landing on the head instead of paws when descending; and 0 for falling off or nearly falling off the ledge, or refusal to move despite encouragement.

#### Open-field test

Mice were placed in the center of a white acrylic chamber (40 × 40 cm) to move freely for 20 min. The movements were tracked and analyzed by mouse-tracking software (EthoVision XT 11.5, Noldus).

#### Neuropathic pain model

PSL or sham surgery was performed on adult mice (7 weeks old). Isoflurane was inhaled to anesthetize mice before and during the surgery. The skin incision was made on the right thigh to expose the sciatic nerve. For the mice in the neuropathic pain group, the right sciatic nerve was partially ligated with 8–0 silk to induce neuropathic pain. In the sham surgery group, an identical procedure was performed, except the sciatic nerve was not ligated. The muscles and skin were closed with 6–0 silk. After surgery, the animals were moved to their cages and monitored during recovery. Mice exhibiting foot drops were excluded from further experiments.

#### Cannula implantation and drug infusion

Mice were anesthetized with an intraperitoneal injection of a mixture of Zoletil and Rompun (30 mg/kg and 10 mg/kg, respectively) and fixed on a stereotaxic frame (Narishige). A small hole was made unilaterally (on the left) at anteroposterior (AP) 1.8 mm, mediolateral (ML) 0.3 mm, and dorsoventral (DV) 1.9 mm from the bregma, which placed the tip of the guide cannula slightly above the injection site. The cannula was then implanted and fixed using dental cement. After the procedure, the mice were returned to their cages and monitored during recovery. After a 1-week recovery, habituation for the von Frey test was performed.

Three weeks later, a volume of 0.5 μl of artificial cerebral spinal fluid (ACSF) or MTEP (15 nmol) was injected into the mPFC through the internal cannula using a syringe pump at a flow rate of 0.1 μl/min with no anesthesia. The internal cannula was kept in place for an additional 2 min after the microinjection to prevent backflow. After the injection, we conducted the behavioral experiment at 30 min after drug injection. This protocol ensured uniform timing and conditions for all experimental subjects.

After all the experiments, mice were anesthetized, and dye was microinjected to confirm the location of the cannula. Mice were perfused with phosphate-buffered saline followed by 4% paraformaldehyde. The brains were extracted, stored in 4% paraformaldehyde, transferred to a 30% sucrose solution and finally sliced into coronal sections using a cryotome.

### Electrophysiology

#### Brain slice preparation

Mice were briefly anesthetized by isoflurane and decapitated. Coronal slices (250 μm thick) were obtained using a vibratome (VT1200S, Leica) with an ice-cold cutting solution containing the following (in mM): 75 sucrose, 75 NaCl, 2.5 KCl, 7 MgCl_2_, 0.5 CaCl_2_, 1.25 NaH_2_PO_4_, 26 NaHCO_3_ and 25 glucose bubbled with 95% O_2_ and 5% CO_2_. The slices were immediately transferred to ACSF containing the following (in mM): 125 NaCl, 2.5 KCl, 1 MgCl_2_, 2 CaCl_2_, 1.25 NaH_2_PO_4_, 26 NaHCO_3_ and 25 glucose bubbled with 95% O_2_ and 5% CO_2_. For recovery, the slices were incubated for 8 min at 32 °C and for an additional 1 h at room temperature. All the recordings were acquired within 8 h after the recovery period.

#### Whole-cell recordings

Coronal brain slices were placed in a submerged chamber and perfused with 32 °C ACSF for at least 10 min before recording. The intracellular solution used in the current clamp was (in mM): 135 K-gluconate, 5 KCl, 2 NaCl, 10 HEPES, 0.1 EGTA, 5 Mg-ATP, 10 phosphocreatine and 0.4 Na_3_-GTP (pH adjusted to 7.2). For the voltage clamp, the following solution was used (in mM): 140 Cs-methanesulfonate, 7 NaCl, 0.2 EGTA, 4 Mg-ATP, 0.3 Na-GTP, 10 HEPES and 5 QX-314. Whole-cell patch-clamp recordings were performed using an patch-clamp amplifier (EPC 9) and Patch Master software (HEKA Elektronik) with a sampling frequency of 20 kHz, and the signals were filtered at 2.9 kHz. All electrophysiological data were acquired in layer 5 of the mPFC. For measuring spontaneous excitatory and inhibitory postsynaptic currents, the membrane potential was held at −70 mV or +10 mV, respectively. The resting membrane potential (RMP) was measured without any holding current in the current clamp mode. Spontaneous inhibitory postsynaptic current (sIPSC) and RMP data were recorded for 3 min. For drug experiments, recordings were obtained at least 15 min after bath application. Any recordings with a series resistance varying by >20% were excluded.

#### Optogenetic manipulation

SST-Ai14 mice were anesthetized with an intraperitoneal injection of a mixture of Zoletil and Rompun (30 mg/kg and 10 mg/kg, respectively) and fixed on a stereotaxic frame (Narishige). A small hole was made at AP 1.8 mm, ML 0.35 mm and DV 1.9 mm from the bregma. For SST interneuron-specific expression, AAV1-EF1ɑ-DIO-eNpHR3.0-EYFP (Addgene #26966) was injected into the prelimbic region of SST-Ai14 mice with a pico pump at 5 nl/s. For slice recording with optogenetic manipulation using an OptoPatcher (A-M systems), a 593.5-nm laser with a power setting of 10 mW and a 2 s duration was used for the current clamp in the SST interneuron. The effect of optogenetic manipulation on sIPSC was confirmed with the same intensity of laser for 10 s.

#### Vector construction and lentivirus injections

A lentiviral vector to overexpress mGluR5 (pLV[FLEXon]-pLV[FLEXon]-EF1A>LL:rev(myc/rat-mGluR5a(ns):T2A:EGFP):rev(LL); VB220510-1907wdb, VectorBuilder, vectorbuilder.com) was packaged through the following steps. Lentiviral particles were produced by co-transfecting lentiviral vector with helper plasmids (psPAX, pMD2.g, from Addgene) into Lenti-X 293T cells (Takara, 632180) grown in DMEM (Gibco, 11965) with 10% fetal bovine serum (Gibco, 10082) and 1% penicillin–streptomycin (GIBCO, 15070), using a polyethylenimine (Polyscience)-based transfection protocol (total DNA-to-polyethylenimine (µg) ratio of 1:2). Plasmids used for virus packaging: 50 µg of lentiviral vector DNA, 37.5 µg of psPAX2 and 12.5 µg of pMD2.g (total 100 µg/245 mm square dish). The medium was replaced 24 h after transfection. Seventy-two hours after transfection, the medium containing lentivirus was centrifuged at 900*g* for 10 min and filtered through a 0.45-µm filter to remove debris. Lentivirus particles were ultracentrifuged at 100,000*g* for 2 h at 4 °C. The concentrated viral pellet was resuspended with sterilized phosphate-buffered saline. The control lentiviral vector (pLV[FLEXon]-EF1A>LL:rev(EGFP):rev(LL); VB220329-1492tph, VectorBuilder) was constructed and packaged by VectorBuilder.

SST-Ai14 mice and SST-cre mice were anesthetized using an intraperitoneal injection of a mixture of Zoletil and Rompun (30 mg/kg and 10 mg/kg, respectively) and fixed on a stereotaxic frame (Narishige). A small hole was made at AP 1.8 mm, ML 0.35 mm and DV 1.9 mm from the bregma. mGluR5 overexpression or control green fluorescent protein (GFP) lentivirus was injected into the left prelimbic region with a pico pump at 5 nl/s. Paw withdrawal thresholds were measured after 1 week of recovery. After finishing all behavior experiments, the brains were extracted, and the injection sites were confirmed.

#### Drugs

All drugs were purchased from Hello Bio. In the whole-cell recordings, 500 μM *R*,*S*-2-chloro-5-hydroxyphenylglycine (CHPG) and 10 μM MTEP were applied to the slices. In the RMP recording shown in Fig. 3, experiments were performed with ACSF containing 10 μM 2,3-dioxo-6-nitro-1,2,3,4-tetrahydrobenzo[f]quinoxaline-7-sulfonamide (NBQX) disodium salt, 30 μM d-2-amino-5-phosphonopentanoate (D-AP5) and 100 μM picrotoxin to block excitatory and inhibitory synaptic inputs.

### Data analysis

Igor Pro 6 (WaveMetrics) was used to analyze whole-cell recording data. Minhee analysis software was used for analyzing sIPSCs^34^. Two-hundred events of sIPSC were selected from each cell and concatenated for comparison of the cumulative fraction.

### Statistical analysis

All statistical data were analyzed using GraphPad Prism, version 9.0 (GraphPad Software). Data for all the experiments are presented as the mean ± standard error of the mean. sIPSCs were compared using an unpaired *t*-test, and the sIPSCs generated after optogenetic manipulation were compared using a paired *t*-test. RMP data for naive mice were analyzed using paired *t*-tests, while RMP data from the sham and pain groups were compared using unpaired *t*-tests. Paw withdrawal thresholds were analyzed using a two-way analysis of variance (ANOVA). A two-sided *P* value less than 0.05 was considered statistically significant.

## Results

### Analgesic effect of an mGluR5 antagonist in the prelimbic area of the mPFC

**Fig. 1:**
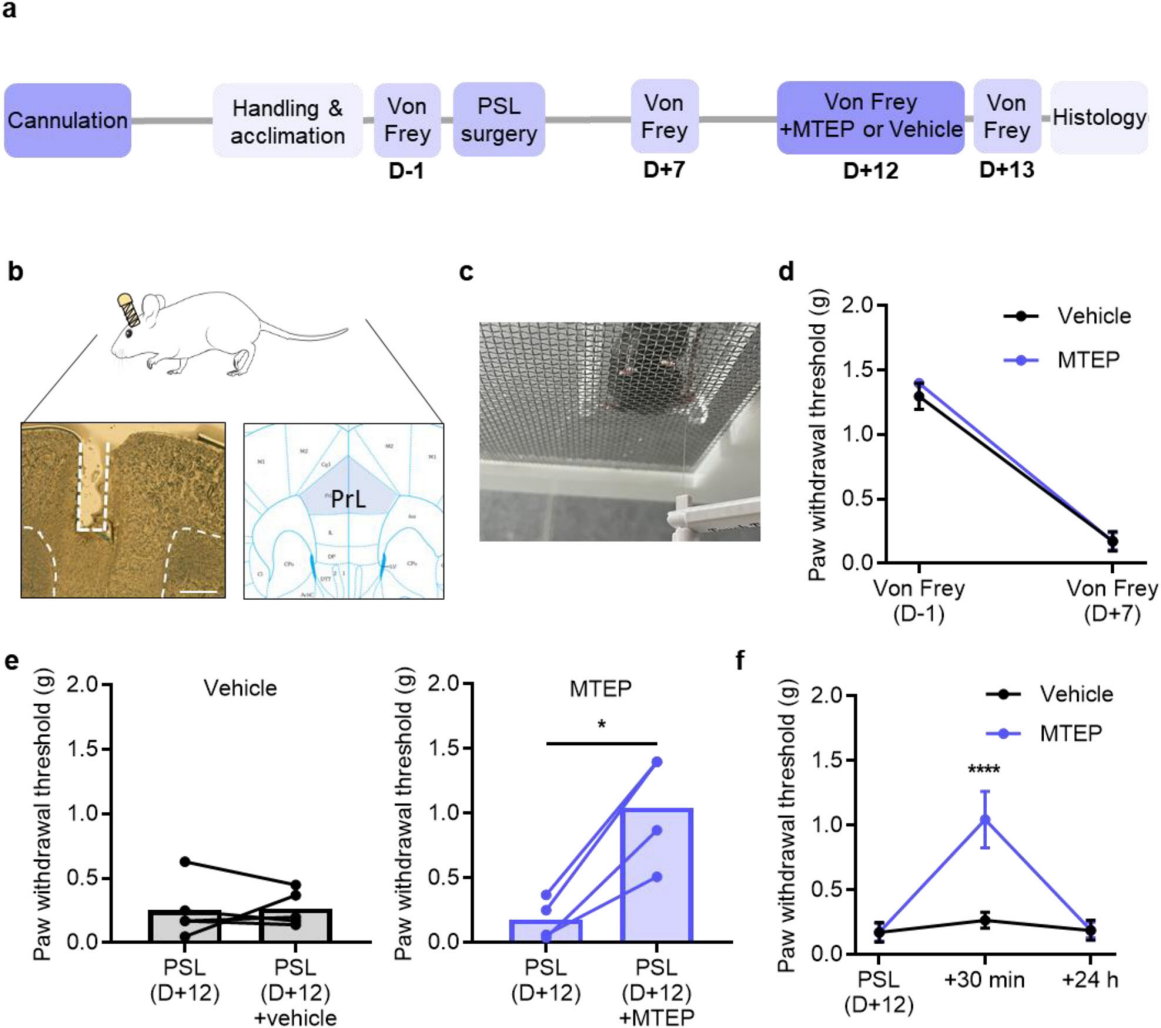
Analgesic effect of an mGluR5 antagonist in the prelimbic area of the mPFC. **a** The experimental schedule for probing the effects of MTEP (an mGluR5 antagonist) on the neuropathic pain model. **b** A representative image of cannula targeting (left) and location of the prelimbic area in brain atlas (right). Scale bar, 500 μm. **c** An image of the von Frey experimental equipment with a mouse taken from the bottom of the mesh. **d** The paw withdrawal threshold before PSL surgery (D−1) and after PSL surgery (D+7) (*n* = 5 mice in the vehicle (black) group, *n* = 4 mice in the MTEP (blue) group; ****P < 0.0001; two-way ANOVA). **e** The paw withdrawal threshold before and 30 min after vehicle (left) or MTEP injection (right) 12 days after PSL surgery (*n* = 5 mice in the vehicle group, *n* = 4 mice in the MTEP group; *P* = 0.8937, *P = 0.0110; paired *t*-test). **f** Paw withdrawal thresholds before surgery and 30 min and 24 h after MTEP injection (*n* = 5 mice in the vehicle group, *n* = 4 mice in the MTEP group; ****P < 0.0001; two-way ANOVA with Bonferroni’s multiple-comparisons test). Data are presented as the mean ± s.e.m. *P < 0.05; ****P < 0.0001.

To investigate the effect of mGluR5 on neuropathic pain in mouse mPFC, we made a neuropathic pain model of PSL surgery and used a pharmacological approach involving a mGluR5 antagonist (MTEP). A cannula was implanted in the left mPFC. After recovery, the mice were tested with the von Frey test to evaluate the baseline withdrawal threshold. PSL surgery was performed on the right side to cause neuropathic pain in the right hind paw (Fig. 1a). We confirmed the cannula location after all experiments were completed (Fig. 1b). To confirm the successful induction of neuropathic pain, the paw withdrawal threshold was measured on day 7 after PSL surgery (Fig. 1c). The paw withdrawal threshold was significantly lower than the threshold before surgery, and these model animals were randomly assigned to the experimental group or control (Fig. 1d). We next sought to see if the deactivation of mGluR5 in the mPFC would exert an analgesic effect. We injected MTEP into the left mPFC 12 days after surgery and measured the paw withdrawal threshold 30 min later. Notably, the injection of MTEP into the mPFC ameliorated the PSL-induced neuropathic pain shown by a significantly increased paw withdrawal threshold (Fig. 1e). This analgesic effect disappeared within 24 h (Fig. 1f). Taken together, these data demonstrate that the administration of the mGluR5 antagonist in the mPFC could alleviate mechanical allodynia, a representative symptom of neuropathic pain.

### Inhibitory input increases in mice with neuropathic pain and can be modulated by MTEP

**Fig. 2:**
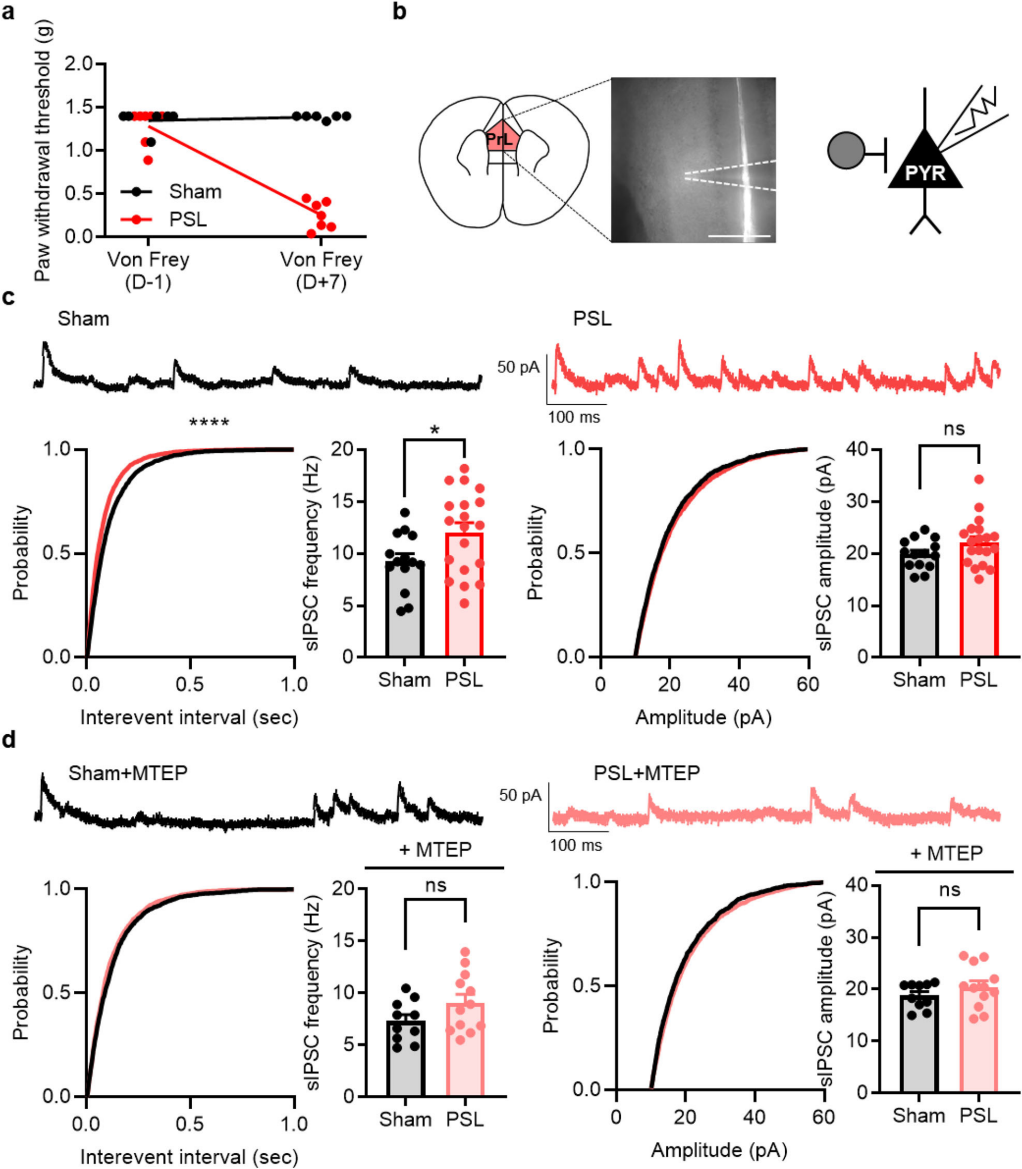
Inhibitory input increases in mice with neuropathic pain and can be modulated by MTEP. **a** Paw withdrawal thresholds before PSL surgery (D−1) and after PSL surgery (D+7) (*n* = 6 mice in the sham group, *n* = 8 mice in the neuropathic pain group; ****P < 0.0001; two-way ANOVA). **b** Left: a schematic illustration of the prelimbic region (left) and representative brain slice image of the prelimbic region of the mPFC (right). Scale bar, 500 μm. Right: a schematic illustration of electrophysiological recording of a pyramidal neuron. **c** Top: representative traces of sIPSC recordings for the sham (black) and neuropathic pain (red) groups. Bottom: the cumulative fraction and average sIPSC frequency (left) and amplitude (right) for the sham and neuropathic pain groups (*n* = 14 cells from 4 mice in the sham group, *n* = 19 cells from 4 mice in the neuropathic pain group; frequency, ****P < 0.0001; amplitude, ns *P* = 0.4595; Kolmogorov–Smirnov test; *P = 0.0307, *P* = 0.1079; unpaired *t*-test). **d** Top: representative traces of sIPSC recordings for the sham (black) and neuropathic pain (light red) groups treated with an MTEP bath application. Bottom: the cumulative fraction and average sIPSC frequency (left) and amplitude (right) for the sham and neuropathic pain groups treated with the MTEP bath application (*n* = 10 cells from 3 mice in the sham group, *n* = 12 cells from 4 mice in the neuropathic pain group; frequency, ns *P* = 0.1294; amplitude, ns *P* = 0.0955; Kolmogorov–Smirnov test; ns *P* = 0.1103, ns *P* = 0.2782; unpaired *t*-test). Data are presented as the mean ± SEM. ns, not significant; *P < 0.05, ****P < 0.0001. PYR, pyramidal neuron.

We conducted patch-clamp recordings to investigate how mGluR5 antagonism affects the local circuitry of the mPFC in mice with neuropathic pain. Because deactivation of the mPFC is a major feature of neuropathic pain and there are reports that inhibitory neurons potentially affect the output of layer 5 pyramidal neurons^12^, we sought to compare the sIPSC of pyramidal neurons in brain slices from sham and neuropathic pain mice. To perform electrophysiological recordings in the neuropathic pain model mice, we confirmed the induction of neuropathic pain through the von Frey test and then made brain slices (Fig. 2a, b). The frequency of the sIPSC in the neuropathic mice was significantly higher than that in the sham mice, whereas the amplitude remained unchanged (Fig. 2c). This indicates that inhibitory tone to the layer 5 pyramidal neurons in the mPFC was enhanced in the neuropathic pain model, suggesting the increased activity of inhibitory neurons.

To determine the possible role of mGluR5 in this change of inhibitory tone in the mPFC, we applied MTEP to the slices through bath application. The increased frequency of the sIPSC in the pyramidal cells from neuropathic mice was diminished by the application of MTEP, returning the sIPSC frequency to the sham control level (Fig. 2d). The level of sIPSC amplitude was unaffected. These results show that mGluR5 plays an important role in the increase in inhibitory input to mPFC layer 5 pyramidal neurons in neuropathic pain model mice.

### Activation of mGluR5 increases inhibitory input to pyramidal neurons

**Fig. 3:**
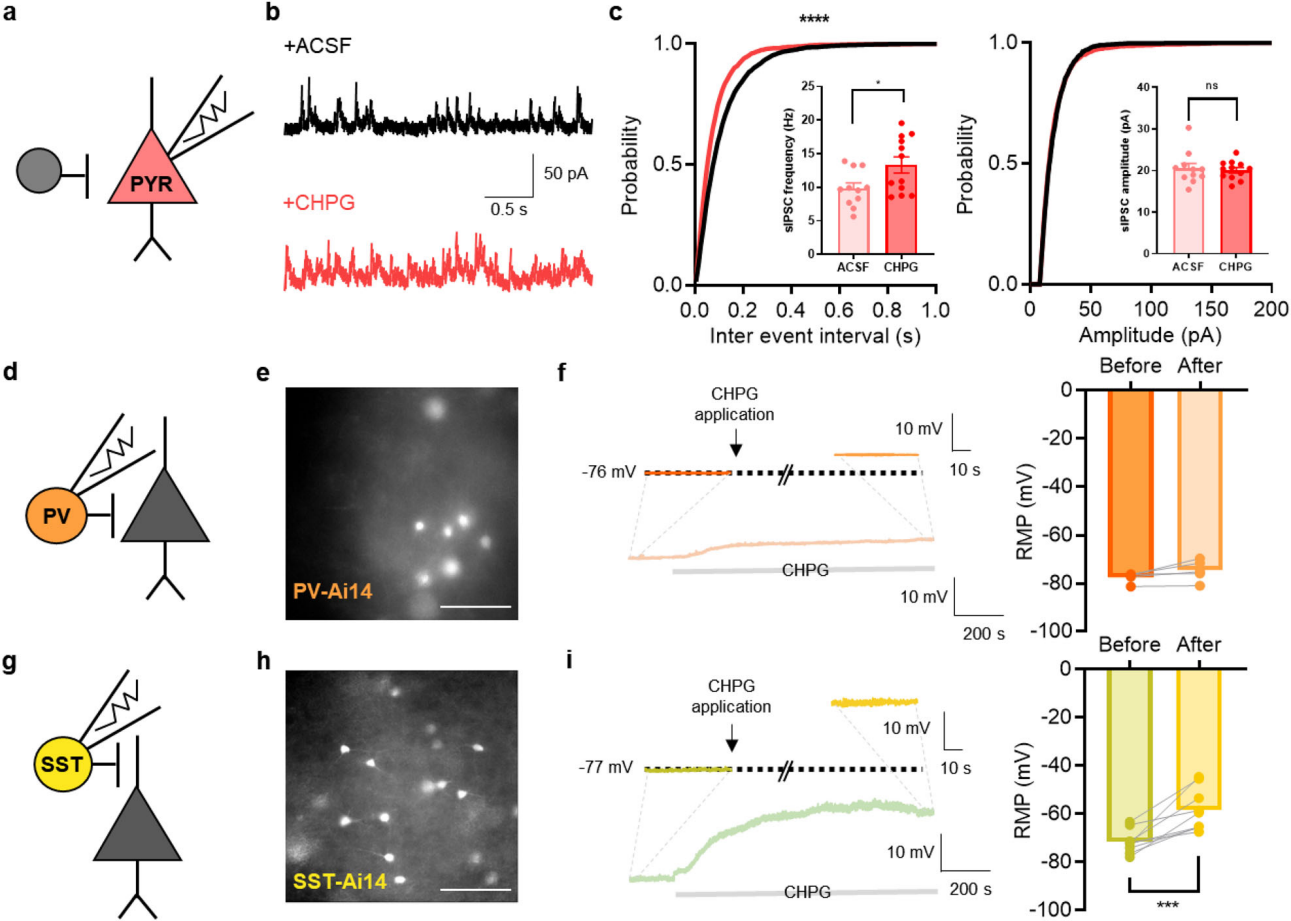
Activation of mGluR5 increases inhibitory input to pyramidal neurons. **a** A schematic illustration of electrophysiological recording of a pyramidal neuron. **b** Representative traces of sIPSC recordings after bath application of ACSF (black) or CHPG (red, mGluR5 agonist) in naive mice. **c** Left: the cumulative fraction of sIPSC frequency and the average sIPSC frequencies with ACSF or CHPG bath application. Right: the cumulative distribution curves of sIPSC amplitude and the average sIPSC amplitude with ACSF or CHPG bath application (*n* = 11 cells from 5 mice in the ACSF group, *n* = 12 cells from 4 mice in the CHPG group; frequency, ****P < 0.0001; amplitude, ns *P* = 0.1596; Kolmogorov‒Smirnov test; *P = 0.0273; unpaired *t*-test). **d** A schematic illustration of electrophysiological recording of PV interneurons. **e** A representative image of td-Tomato-expressing PV interneurons used for electrophysiological recording. Scale bar, 100 μm. **f** Top: representative traces of RMP before and after CHPG application to PV interneurons. Bottom: a representative trace of the entire time course of CHPG effect. Quantification of the average RMP before and after CHPG application (*n* = 5 cells from 3 mice; *P* = 0.0863; paired *t*-test). **g** A schematic illustration of electrophysiological recording of SST interneurons. **h** A representative image of td-Tomato-expressing SST interneurons used for electrophysiological recording. Scale bar, 100 μm. **i** Top: representative traces of the RMP before and after CHPG application to SST interneurons. Bottom: a representative trace of the entire time course of CHPG effect. Quantification of the average RMP before and after CHPG application (*n* = 9 cells from 5 mice; ***P = 0.0009; paired *t*-test). Data are presented as the mean ± s.e.m. ns, not significant. *P < 0.05, ***P < 0.001, ****P < 0.0001.

We recorded the sIPSC in layer 5 pyramidal neurons following the application of CHPG, an mGluR5 agonist, to brain slices from naive mice (Fig. 3a). The application of CHPG significantly increased the frequency of sIPSC, making it comparable to the neuropathic pain state. This result demonstrates that the mGluR5 activation in the mPFC area reinforces inhibitory tone to layer 5 pyramidal neurons, suggesting that a large proportion of mPFC output would be inhibited by this change. The amplitude of the sIPSC was not altered by CHPG, suggesting the existence of a presynaptic mechanism involving mGluR5 receptors that act on inhibitory interneurons (Fig. 3b, c).

Next, we sought to identify the types of interneuron involved in the mGluR5-mediated increase in sIPSC frequency observed in pyramidal neurons. It is well known that two types of interneuron inhibit pyramidal neurons in the prefrontal cortex: PV-expressing interneurons and SST-expressing interneurons. To identify each cell type, we bred mutant mice that expressed td-Tomato in PV or SST interneurons (Fig. 3d, e, g, h). Given that mGluR5 activation depolarizes the membrane potential of cells, we compared the RMP of each type of interneuron with the administration of CHPG. We found that only the SST interneurons were significantly depolarized by CHPG treatment. The PV interneurons were also slightly depolarized, but the change was not significant (Fig. 3f, i). Through these results, we found that the cell type that is strongly affected by mGluR5 in the mPFC is the SST interneuron.

### Neuropathic pain depolarizes SST interneurons via mGluR5 activation

**Fig. 4:**
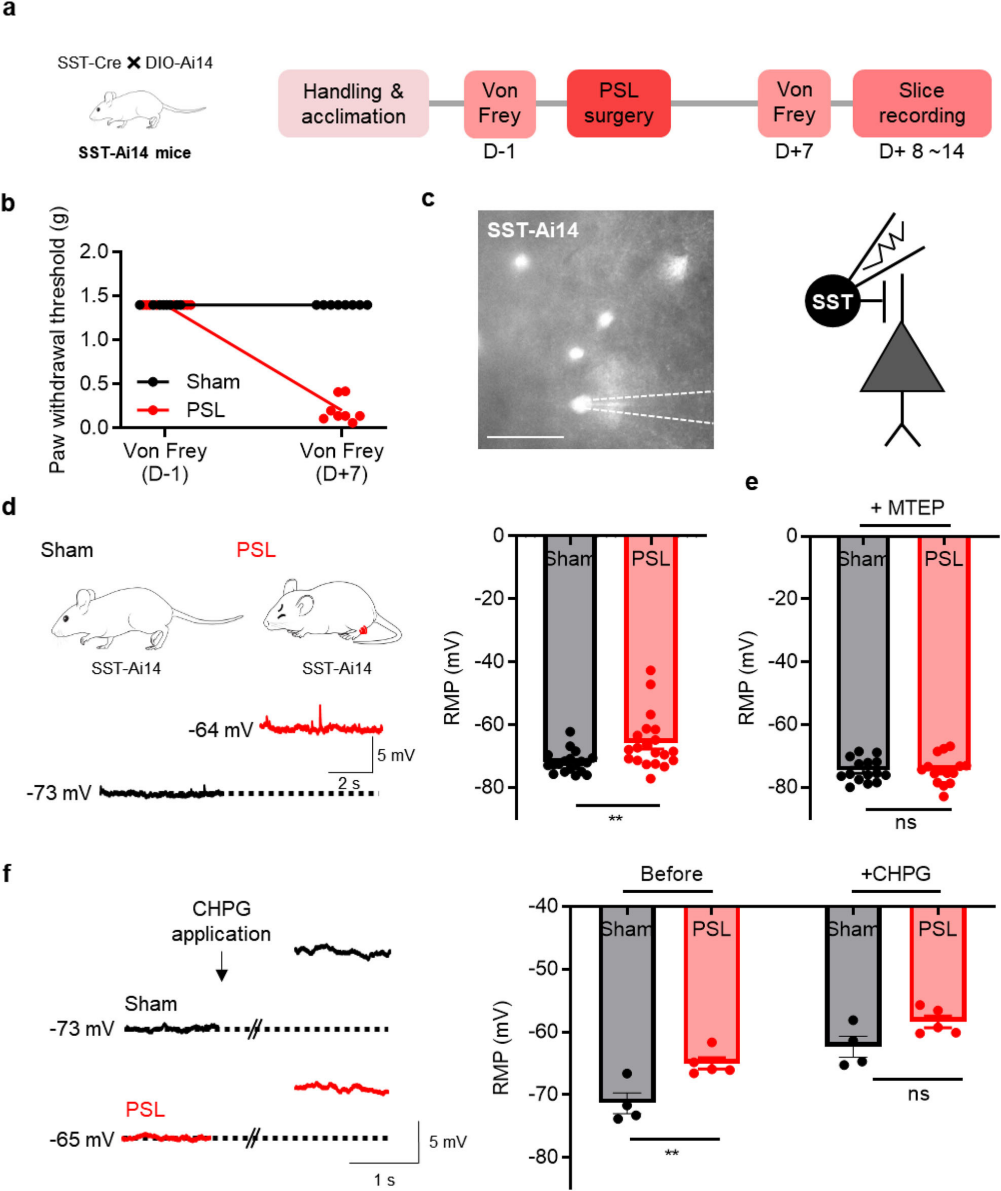
Neuropathic pain depolarizes SST interneurons via mGluR5 activation. **a** A schematic image of SST-Ai14 mice and the experimental schedule for slice recording in the neuropathic model. **b** Paw withdrawal thresholds before PSL surgery (D−1) and after PSL surgery (D+7) (*n* = 7 mice in the sham group, *n* = 8 mice in the neuropathic pain group; ****P < 0.0001; two-way ANOVA). **c** Left: a representative image of td-Tomato-expressing SST interneurons with electrophysiological recording. Scale bar, 50 μm. Right: a schematic illustration of electrophysiological recording of SST interneurons. The dotted lines indicate the position of the patch pipette. **d** Representative traces of the RMP in the sham (left) and neuropathic pain groups (right). Quantification of the average RMP in the sham (black; *n* = 19 cells from 3 mice in the sham group) and neuropathic pain groups (red; *n* = 20 cells from 3 mice in the neuropathic pain group; **P = 0.0061; unpaired *t*-test). **e** Quantification of the average RMP in the sham (black; *n* = 15 cells from 2 mice in the sham group) and neuropathic pain groups with MTEP application (red; *n* = 15 cells from 2 mice in the neuropathic pain group; ns P = 0.9704; unpaired *t*-test). **f** Representative traces of the RMP in the sham (black) and neuropathic pain (red) groups before and after CHPG (mGluR5 agonist) application. The average RMP for the sham (black) and neuropathic pain (red) groups before and after CHPG application (*n* = 4 cells from 4 mice in the sham group, *n* = 5 cells from 5 mice in the neuropathic pain group; sham and neuropathic pain group before CHPG application, **P = 0.0062; sham and neuropathic pain group after CHPG application, ns *P* = 0.0809; two-way ANOVA). Data are presented as the mean ± s.e.m. ns, not significant; **P < 0.01, ****P < 0.0001.

Based on the above experiments, we hypothesized that the neuropathic pain-induced upregulation of mGluR5 occurs in SST interneurons leading to increased inhibitory tone to pyramidal neurons. Given that SST interneurons were significantly depolarized by CHPG treatment, changes in the membrane potential of these interneurons would be observed in the neuropathic pain state if the mGluR5 upregulation occurred. To record the membrane potential of SST interneurons in neuropathic pain model mice, we induced neuropathic pain through PSL surgery in mutant mice expressing td-Tomato in SST interneurons (Fig. 4a). We confirmed the formation of pain through the von Frey test (Fig. 4b). The RMP of the SST interneurons in the pain model mice was significantly higher than that in the sham model mice (Fig. 4c, d). To investigate whether the observed significant difference was potentially related to mGluR5 upregulation, we examined the RMP after MTEP treatment. Interestingly, we found that the significant difference between groups was no longer present after MTEP application. This observation strongly implies mGluR5 involvement in the observed RMP changes (Fig. 4e). We further hypothesized that the mGluR5 activation would bring RMP levels in the sham group to that of the neuropathic pain group. To test this, we applied CHPG to brain slices from the neuropathic pain and sham groups through bath application during RMP recordings. As we expected, the CHPG treatment depolarized the SST interneurons, bringing the RMP of the sham group to the level of the neuropathic pain group. Consequently, the difference in RMP of the SST interneurons between the neuropathic pain and sham group was diminished (Fig. 4f). Input resistance was not significantly different between groups (Supplementary Fig. 2). Furthermore, we extended our investigation to compare the effects of CHPG on PV interneurons. Unlike SST interneurons, PV interneurons exhibited neither pain-induced differences nor changes in intergroup differences in response to CHPG treatment (Supplementary Fig. 1a–c). Our findings show that mGluR5 activity in the mPFC is involved in the depolarization of the SST interneurons in the neuropathic pain state.

### Optogenetic inhibition of SST interneurons reduces the enhanced sIPSC in the neuropathic pain model

**Fig. 5:**
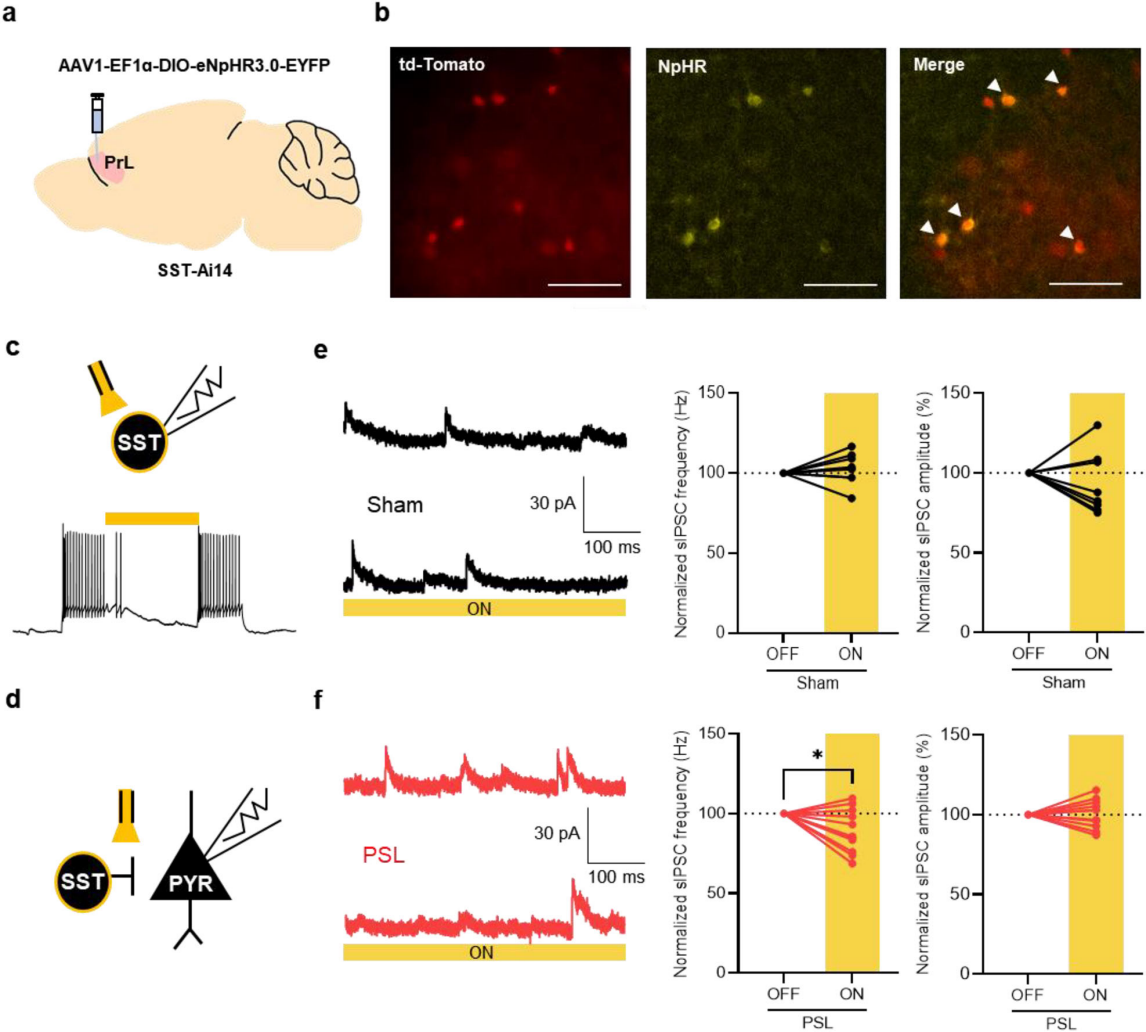
Optogenetic inhibition of SST interneurons reduces the enhanced sIPSC in the neuropathic pain model. **a** A schematic illustration of in vivo virus injection for SST interneuron specific optogenetic modulation in the prelimbic region of the mPFC. **b** Representative images of td-Tomato (left), NpHR (middle) and merge (right) expression in the SST interneurons. Scale bar, 100 μm. **c** Top: a schematic illustration of electrophysiological recording of SST interneurons with NpHR virus expression. Bottom: a representative trace of current clamp recording in SST interneurons expressing NpHR. The yellow bar indicates the period of 593-nm laser illumination. **d** A schematic illustration of the electrophysiological recording of pyramidal neurons with NpHR virus expression in SST interneurons. **e** Left: representative traces of sIPSC recordings before (top) and during laser illumination (bottom) in the sham group. Right: quantification of averaged frequency (left) and amplitude (right) of the sIPSC before and during 593-nm laser illumination in the sham (black) group (*n* = 8 cells from 2 mice in the sham group; *P* = 0.4634, *P* = 0.3710; paired *t*-test). **f** Left: representative traces of sIPSC recordings before (top) and during laser illumination (bottom) in the neuropathic pain group. Right: quantification of averaged frequency (left) and amplitude (right) of the sIPSC before and during 593-nm laser illumination in the neuropathic pain (red) group (*n* = 11 cells from 2 mice in the neuropathic pain group; *P = 0.0255, *P* = 0.6434; paired *t*-test). Data are presented as the mean ± s.e.m. *P < 0.05; PYR, pyramidal neuron.

Furthermore, we performed cell-type-specific optogenetic experiments to determine whether the increase in inhibitory input of neuropathic pain mice is derived from SST interneurons. To specifically modulate the activity of SST interneurons, we injected a virus expressing the inhibitory chloride pump *Natronomonas* halorhodopsin (NpHR) in a Cre-dependent manner into SST-Ai14 mice (Fig. 5a, b). Next, we confirmed the functionality of NpHR virus by demonstrating reduced excitability through laser illumination (Fig. 5c). In the brain slice of sham and neuropathic pain groups, sIPSC was recorded in the pyramidal neurons with optogenetic inhibition of SST interneurons (Fig. 5d). In the sham group, optogenetic suppression of SST interneurons did not induce a significant change in sIPSCs of pyramidal neurons (Fig. 5e). Only in the neuropathic pain group, in which the sIPSC frequency of pyramidal neurons had been increased, was sIPSC frequency significantly decreased when the activity of the SST interneuron was suppressed by optogenetic manipulation (Fig. 5f and Supplementary Fig. 3). These results demonstrate that the activity of SST interneurons in the mPFC is a key mechanism underlying the increased sIPSC frequency of pyramidal neurons in the neuropathic pain state.

### Lentiviral overexpression of mGluR5 in SST interneurons induces mechanical allodynia

**Fig. 6:**
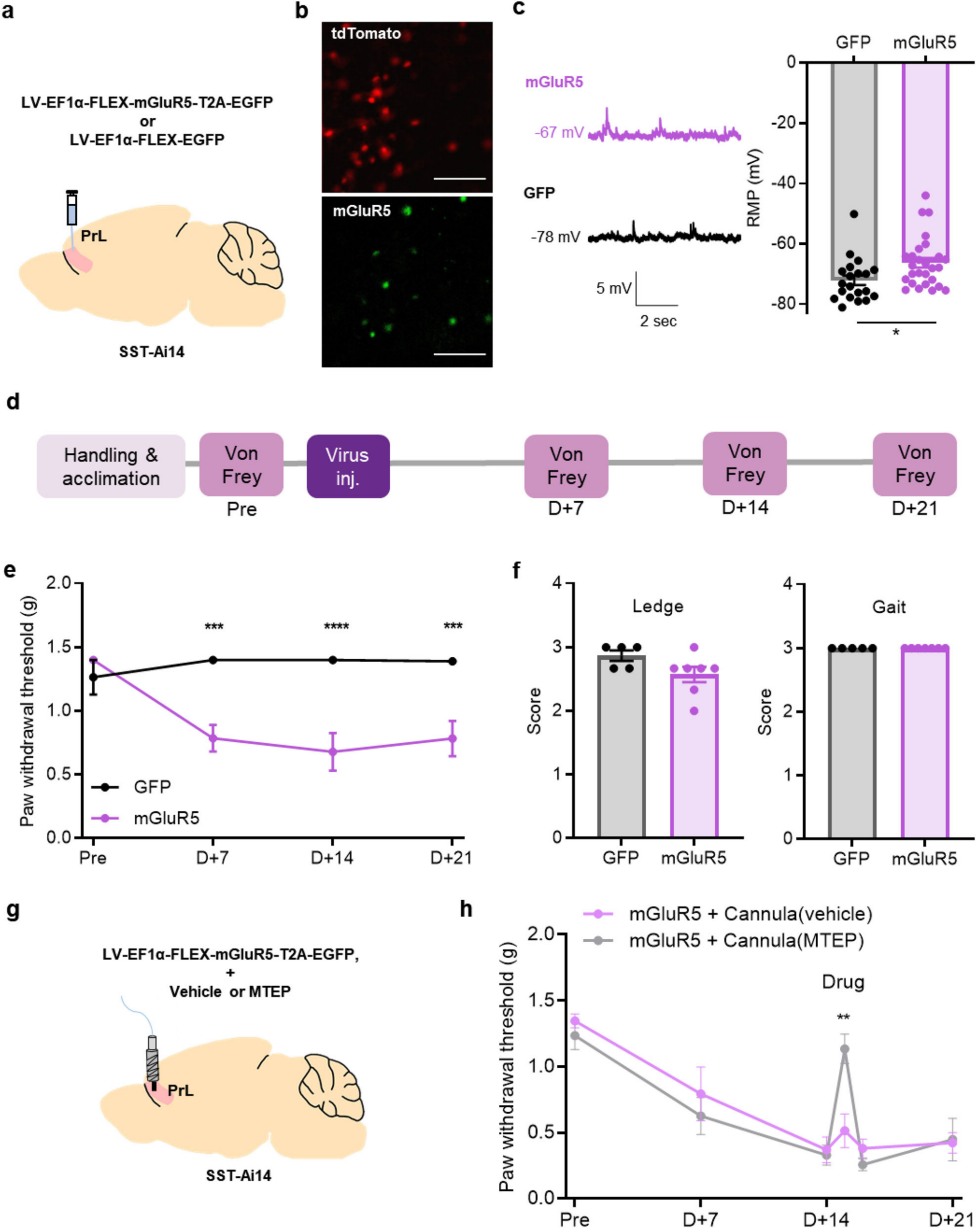
Lentiviral overexpression of mGluR5 in SST interneurons induces mechanical allodynia. **a** A schematic illustration of in vivo virus injection for SST interneuron-specific mGluR5 modulation in the prelimbic region of the mPFC. **b** A representative image of td-Tomato expression (top) and LV-EF1α-FLEX-mGluR5-T2A-GFP expression in mPFC of SST-Ai14 mice (bottom). Scale bar, 100 μm. **c** Representative traces of the RMP in the mGluR5 (purple) and GFP expressing (black) cells. The average RMP in LV-EF1α-FLEX-EGFP (GFP) and LV-EF1α-FLEX-mGluR5-T2A-GFP (mGluR5)-expressing SST interneurons (*n* = 20 cells from 4 mice in the GFP group; *n* = 27 cells from 4 mice in the mGluR5 group; *P = 0.0155; unpaired *t*-test). **d** The experimental schedule for the pain behavior test in virus-injected mice. **e** Paw withdrawal thresholds before and 7, 14 and 21 days after virus injection (*n* = 5 mice in the GFP group; *n* = 7 mice in the mGluR5 group; ***P = 0.0007 on day 7; ****P < 0.0001 on day 14; ***P = 0.0008 on day 21; two-way ANOVA). **f** Quantification of the ledge and gait test scores for GFP- and mGluR5-expressing mice (*n* = 5 mice in the GFP group, *n* = 7 mice in the mGluR5 group; *P* = 0.0928 in the ledge test). **g** A schematic illustration of cannulation and in vivo virus injection for SST interneuron specific mGluR5 modulation in the prelimbic region of the mPFC. **h** Paw withdrawal thresholds before and 7, 14, 15, 16 and 21 days after virus injection. MTEP was injected on D+15 before the von Frey test (*n* = 6 mice in the vehicle group; *n* = 5 mice in the MTEP group; **P = 0.0024 on day 15; two-way ANOVA). Data are presented as the mean ± s.e.m. *P < 0.05, ***P < 0.001, ****P < 0.0001.

Finally, to determine whether SST interneuron-specific mGluR5 regulation affects nociception, we injected Cre-dependent mGluR5-overexpressing lentivirus into SST-Ai14 mice to overexpress mGluR5 specifically in SST interneurons (Fig. 6a). Before patch-clamp recording, we checked brain slices for td-Tomato fluorescence to confirm the presence of SST interneurons and for GFP to confirm viral infection (Fig. 6b). A comparison of the RMP of cells expressing GFP in the control-GFP and mGluR5 groups revealed that mGluR5 overexpression can result in sustained cell depolarization (Fig. 6c). We hypothesized that, similar to the mGluR5 increase induced by PSL, genetic manipulation could induce neuropathic pain symptoms. Therefore, we measured the paw withdrawal threshold of the mice in the SST interneuron-specific mGluR5-overexpression group using the von Frey test (Fig. 6d). Remarkably, the mGluR5 group showed a lower paw withdrawal threshold than the GFP group, even though they had not undergone PSL surgery (Fig. 6e). There were no significant differences between the GFP and mGluR5 groups in the gait, ledge and open-field tests (Fig. 6f and Supplementary Fig. 4a). These data demonstrated that SST interneuron-specific mGluR5 genetic manipulation can induce mechanical allodynia, a representative symptom of neuropathic pain. To further investigate whether the allodynia induced by mGluR5 overexpression could be alleviated by an mGluR5 antagonist, similar to the allodynia reduction observed in the neuropathic pain group treated with MTEP, we conducted additional experiments. After viral expression, we administered MTEP through cannula injection (Fig. 6g). This treatment resulted in an increased pain threshold, demonstrating that MTEP effectively attenuated the virus-induced allodynia (Fig. 6h). An open-field test revealed no differences between groups, indicating that the observed effects were specific to allodynia without affecting general locomotion (Supplementary Fig. 4b). This result suggests that mGluR5 in SST interneurons is a potential pharmacotherapeutic target to prevent neuropathic pain development and treat the already established symptoms.

## Discussion

In a previous study of ours, we reported that the upregulation of mGluR5 in mPFC plays an important role in neuropathic pain symptoms. Likewise, other studies have reported that reduced activity in the mPFC is associated with neuropathic pain symptoms. In this regard, it remains unexplained how the mPFC activity is decreased in the neuropathic pain state, despite increased mGluR5 activation. Therefore, we aimed to find mechanisms by which decreased mPFC activity is accompanied by mGluR5 activation in the neuropathic pain state. First, we verified that the application of an mGluR5 antagonist in the mPFC induces an analgesic effect and rescues the increased sIPSC frequency in pyramidal neurons in the context of neuropathic pain. Then, we demonstrated that an mGluR5 agonist increases the inhibitory inputs to layer 5 pyramidal neurons and that this effect is mediated mainly by the depolarization of SST interneurons. Likewise, the neuropathic pain model mice showed an increase in the frequency of the sIPSC in layer 5 pyramidal neurons; this effect was mediated by mGluR5-dependent SST interneuron depolarization and could be reversed by optogenetic inactivation of SST interneurons. Finally, we discovered that upregulation of mGluR5 in SST interneurons causes mechanical allodynia. These findings show that mGluR5 in SST interneurons is activated in the neuropathic pain state and that enhanced inhibition of pyramidal neurons is responsible for neuropathic pain.

Previously, we reported mGluR5 alteration in the brain of the neuropathic pain model through positron emission tomography imaging^29,30^. In particular, mGluR5 was shown to be upregulated in the mPFC and to play an important role in modulating neuropathic pain in rats^30^. In the current study, we confirmed this effect in mice through a pharmacological approach. This shows that mGluR5 plays an important role in modulating neuropathic pain in mice, and the same trends are observed across species. Based on this, we believe that mGluR5 will behave in the same way in more species, especially in humans. Regarding this analgesic effect, treatment with an mGluR5 antagonist rescued the same changes seen in the neuropathic pain situation both in vivo and ex vivo. Electrophysiological data showed that treatment with an mGluR5 antagonist reduced inhibitory signaling in the neuropathic pain model, suggesting that the analgesic effect of in vivo was mediated by reducing the enhanced inhibitory signaling in the mPFC.

Furthermore, using electrophysiological recordings, we elucidated that SST interneurons in the mPFC are particularly important for pain processing changes in the neuropathic pain state. Our results demonstrate that alterations in mGluR5 in SST interneurons play an important role in enhanced inhibition to pyramidal neurons. Previous studies have focused only on the importance of PV interneurons in the mPFC for the establishment of neuropathic pain models^14,32,34^. Optogenetic modulation of PV interneurons changes pain thresholds in mice, and input from the basolateral amygdala to PV interneurons plays a role in neuropathic pain mice^14,34^. In this study, we suggest that SST interneurons also play an important role in neuropathic pain. It remains to be investigated how these two types of interneuron work in the same direction with different mechanisms.

In this study, we focused on the local circuit of the mPFC, a key area of the pain circuitry that receives input from many different areas and sends out output signals^11,15,35^. For example, the mPFC receives inputs from the basolateral amygdala^15^ and sends output to the periaqueductal gray^10^. The extent of the input and output may vary across the cell type in the mPFC. In terms of inputs, it is necessary to determine whether such pain-related brain areas directly project to SST interneurons in the mPFC and how they change in the context of neuropathic pain. Future studies focusing on changes in input signals may provide insight into the specific mechanisms by which mGluR5 is increased in SST interneurons during the development of neuropathic pain. Regarding the outputs, the result that mGluR5 overexpression in SST interneurons causes mechanical allodynia suggests that this modulation eventually affects subsequent pain circuits, such as the periaqueductal gray, which are involved in pain response behavior^13,15,36^. Therefore, it is important to investigate not only the changes within the mPFC but also the changes in the input and output signals that influence or are influenced by the mPFC.

We measured the RMP to show that SST interneurons are depolarized by mGluR5 activation in the neuropathic pain model. mGluR5 activation can depolarize neuronal activity with several mechanisms. These include changes in ion channels dependent on protein kinase C and activation of nonselective cationic channels such as TRPC channel^37,38^. Our approach to measuring RMP has the advantage of investigating steady states of neurons rather than stimulus-evoked transient changes. The limitation was that we could not compare differences in SST interneuron excitability between neuropathic pain and control owing to the heterogeneous properties of SST interneurons^39,40^. Contrary to our report that SST interneurons were depolarized in neuropathic pain, a few papers have reported no difference in the excitability of SST interneurons in layer 5 before and after pain^6,10^. This discrepancy might be due to RMP being corrected in the process of measuring excitability. If the RMP was corrected to the same level by current injection in measures of intrinsic excitability, the effects of the mGluR5-related depolarization seen in our study would have been attenuated.

An issue that has not yet been fully elucidated in this study is how the observed increase in mGluR5 in the neuropathic pain state exerts its persistent effects in SST neurons. The analgesic effect resulting from MTEP treatment in the mPFC of neuropathic pain model animals demonstrated that, although mGluR5 is broadly expressed in mPFC neurons, the influence of mGluR5 deactivation in inhibitory neurons outweighs the mGluR5 deactivation in excitatory neurons. This also suggests that the mGluR5 in inhibitory neurons may be persistently active in neuropathic pain and, thus, significantly influenced by MTEP. Similarly, mGluR5-overexpression experiments in the naive animals demonstrated persistent upregulation of RMP in SST neurons of the mPFC, with animals exhibiting neuropathic pain-like symptoms. These results support the idea that, in the presence of neuropathic pain, SST neurons in the mPFC neural circuit are under the persistent influence of increased mGluR5. Our interpretation of how the increase in mGluR5 observed in neuropathic pain or induced by viral overexpression produces persistent effects is as follows. (1) Upregulation of mGluR5 levels in the mPFC SST neurons increases the likelihood of mGluR5 activity by either excitatory signaling or ligand-independent constitutive activation. (2) Once mGluR5 is activated, phosphorylation of intracellular molecules involved in mGluR5 signaling occurs, leading to a relatively depolarized RMP and a sustained mGluR5 activation in SST neurons. (3) The inhibition of SST neurons on pyramidal neurons increases, resulting in a larger pain signal being transmitted to the mPFC. This, in turn, may increases glutamate concentration in the synaptic cleft, leading to a high possibility of persistent mGluR5 activation in SST neurons.

Our study advances our understanding of which cell types in the mPFC mGluR5 are activated in the neuropathic pain state. We suggest that SST interneurons are an important cell type that mediates neuropathic pain and that upregulation of mGluR5 in SST interneurons in the mPFC is responsible for neuropathic pain development. The identification of specific molecules in specific cells in neuropathic pain situations will provide a basis for the development of targeted pharmacotherapeutic strategies. Future studies would show a potential therapeutic strategy for pain treatment through SST interneuron-specific mGluR5 modulation.

## Supplementary information

Supplementary Information
