# Supplementary material for: Increased mGluR5 in somatostatin-positive interneurons mediates deactivation of the mPFC in a mouse model of neuropathic pain
Source: Exp Mol Med. 2025 Apr 10;57(4):888–99. doi: 10.1038/s12276-025-01435-y (PMC12046054; doi:10.1038/s12276-025-01435-y)
Supplement: Supplementary file 1 — Supplementary Information [file 12276_2025_1435_MOESM1_ESM.pdf]

Supplementary information

Increased mGluR5 in somatostatin-positive interneurons mediates deactivation of the mPFC in a mouse model of neuropathic pain

Supplementary Figures

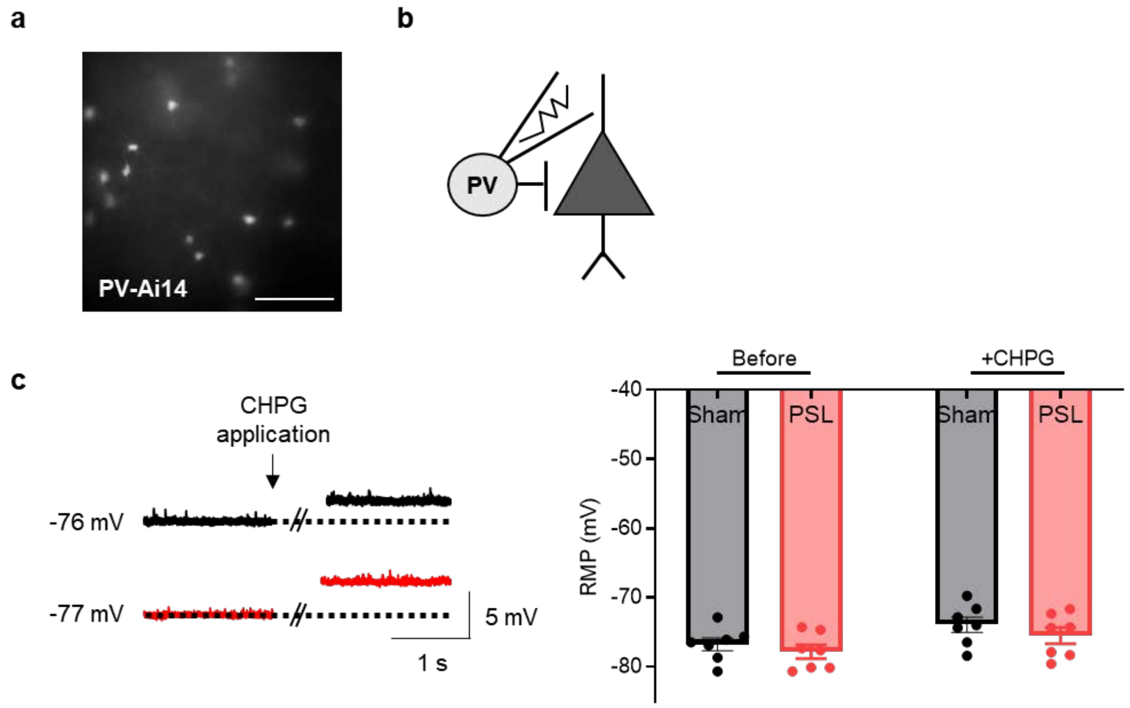

Supplementary Fig. 1. PV interneuron are not affected by neuropathic pain-induced mGluR5 upregulation.

(a) Representative image of td-Tomato-expressing PV interneurons with electrophysiological recording. Scale bar, 100 μm. **(b)** Schematic illustration of electrophysiological recording of PV interneuron. **(c)** Representative traces of the RMP in the sham (black) and neuropathic pain (red) groups before and after CHPG (mGluR5 agonist) application. The average RMP for the sham (black) and neuropathic pain (red) groups before and after CHPG application (n = 7 cells from 4 mice in the sham group, n = 7 cells from 3 mice in the neuropathic pain group; sham and neuropathic pain group before CHPG application, n.s. p = 0.7247; sham and neuropathic pain group after CHPG application, n.s. p = 0.5110; two-way ANOVA). Data are presented as the mean ± SEM. n.s. not significant; PV: parvalbumin, RMP: resting membrane potential.

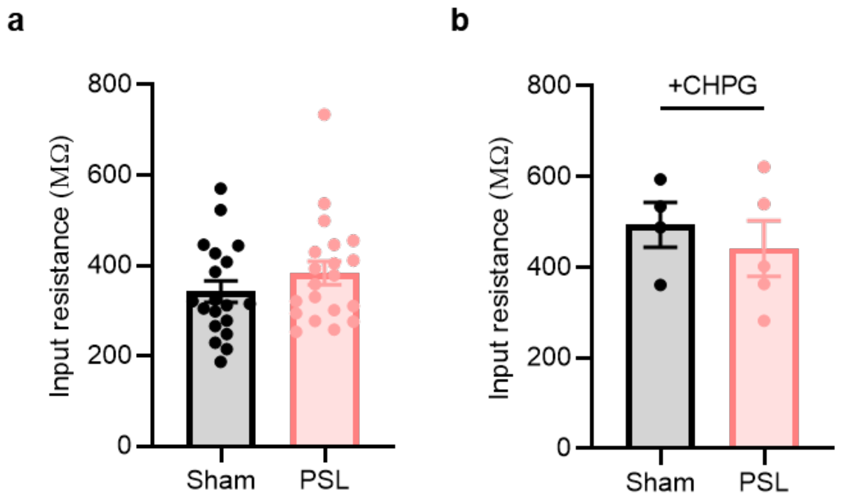

Supplementary Fig. 2. No differences in input resistance observed across pain states and CHPG treatment conditions.

(a) Input resistance in the sham (black) and neuropathic pain (pink) groups (n = 19 cells from 3 mice in the sham group; n = 20 cells from 3 mice in the neuropathic pain group; n.s. = 0.2541; unpaired t test). (b) Input resistance in the sham (black) and neuropathic pain (pink) groups with CHPG application (n = 4 cells from 4 mice in the sham group; n = 5 cells from 5 mice in the neuropathic pain group; n.s. = 0.5431; unpaired t test). Data are presented as the mean ± SEM. n.s. not significant.

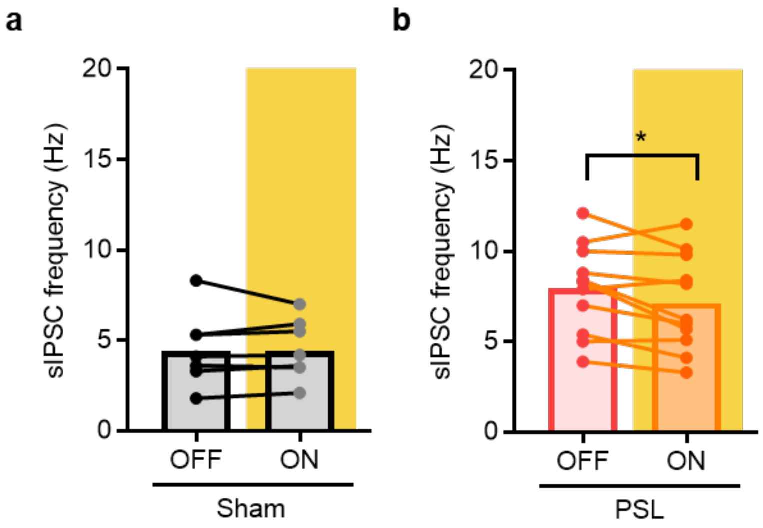

Supplementary Fig. 3. Inhibition of SST interneurons affects the sIPSC only in the neuropathic pain model.

(a) The averaged frequency of the sIPSC before and during 593 nm laser illumination in the sham (black) group (n =8 cells from 2 mice in the sham group; n.s p > 0.9999; paired t test). (b) The averaged frequency of the sIPSC before and during 593 nm laser illumination in the neuropathic pain (red) group (n = 11 cells from 2 mice in the neuropathic pain group; * p = 0.0411; paired t test). Data are presented as the mean ± SEM. n.s. not significant; * p < 0.05; sIPSC: spontaneous inhibitory postsynaptic current, SST: somatostatin.

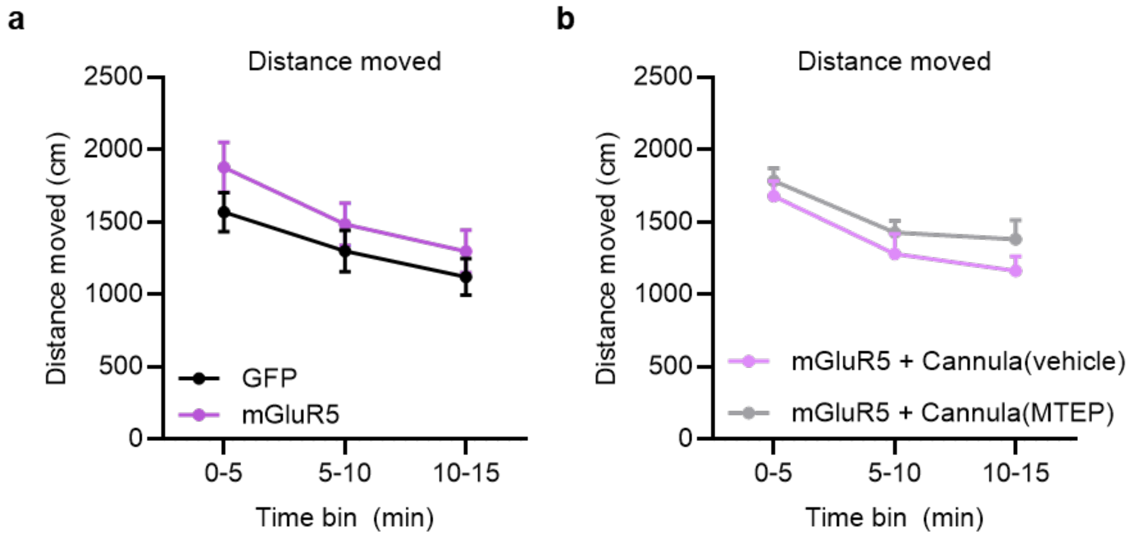

Supplementary Fig. 4. mGluR5 overexpression does not affect general locomotor activity.

(a) Total distance moved by GFP and mGluR5 expressing mice (n = 5 mice in the GFP group, n= 7 mice in the mGluR5 group; n.s. = 0.6663; two-way ANOVA). (b) Total distance moved by mGluR5 expressing mice with vehicle or MTEP injection (n = 6 mice in the vehicle group, n= 5 mice in the MTEP group; n.s. = 0.7307; two-way ANOVA). Data are presented as the mean ± SEM. n.s. not significant; mGluR5: metabotropic glutamate receptor 5, GFP: green fluorescent protein.
